# Are microRNAs located in genomic regions associated with cancer?

**DOI:** 10.1038/sj.bjc.6603381

**Published:** 2006-09-26

**Authors:** P Lamy, C L Andersen, L Dyrskjot, N Torring, T Orntoft, C Wiuf

**Affiliations:** 1 Bioinformatics Research Center (BiRC), University of Aarhus, Hoegh-Guldbergs Gade 10, Bldg 1090, 8000 Aarhus C, Denmark; 2 Molecular Diagnostic Laboratory, Aarhus University Hospital, Skejby, Brendstrupgaardsvej 100, 8200 Aarhus N, Denmark

**Keywords:** microRNA, affymetrix SNP array, copy number, colon, prostate, bladder

## Abstract

We report on the location of 283 miRNAs in the human genome in relation to copy number changes in three distinct types of tumours: prostate, bladder and colon. In prostate and colon tumours, we find miRNAs over-represented in regions with copy number gain and under-represented in regions with copy number loss. Surprisingly this pattern appears to be reversed in bladder cancer. We compared our miRNA copy number data to published miRNA expression data; unexpectedly, we did not find a statistically significant relationship between miRNA copy number and expression level. This suggests that miRNA expression is regulated through different mechanisms than mRNA expression.

MicroRNAs (miRNAs) are small non-coding RNAs that are conserved in sequences between distantly related organisms. Mature miRNAs (∼22 nucleotides) regulate gene expression by targeting mRNAs for cleavage or translational inhibition (Bartel, 2004). They are known to be differently expressed in different tissues. Moreover, several studies have suggested that miRNAs might be involved in human tumorigenesis (Lu *et al*, 2005; Volinia *et al*, 2006). Calin *et al* (2004) showed that miRNAs are frequently located near genomic fragile sites as well as in cancer-associated genomic regions. They screened PubMed for papers reporting any cancer-related abnormalities and found that 98 of 186 miRNAs were located in these regions. However, their study was not cancer type specific and whether it generalises to specific cancers was not discussed.

Lu *et al* (2005) reported on miRNAs expression profiles (217 miRNAs in 334 samples) and found that the majority of the differentially expressed miRNAs were downregulated in tumour samples compared to normal samples. More recently, Volinia *et al* (2006) showed that miRNAs differently expressed in solid cancer were mostly overexpressed. They evaluated the expression profiles of 228 human miRNAs in 540 samples from six solid tumour types. Based on these, they identified six tissue-specific cancer signatures and proposed that the most common miRNA event in solid tumours is gain of expression, while loss of expression is more tissue-specific and less common.

In this study, we report on the location of miRNAs (*n*=283) in relation to copy number alterations in three specific cancer types, prostate, bladder and colon. We use Affymetrix 10 and 50 k SNP arrays to identify genomic regions with abnormal copy numbers. Further, we discuss our results in relation to the findings of Volinia *et al* (2006) and Lu *et al* (2005).

## MATERIALS AND METHODS

The GeneChipMapping 10 k Early Access Array from Affymetrix was applied to a set of 128 samples (113 normal samples and 15 colon adenocarcinomas) and the GeneChipMapping 50 K array was applied to 143 samples (72 normal samples, 41 prostate tumours and 30 bladder tumours). SNP intensities were extracted using the dChip software and subsequently all intensities were normalised to facilitate comparisons between SNPs and arrays, as described in Supplementary Material. Using this procedure, the intensities in normal samples would be centred on zero with a standard deviation (s.d.) of 1. We defined seven groups of tumours: all prostate tumours (P1), prostate tumours without metastasis (P2) or with metastasis (P3), all bladder tumours (B1), bladder tumours in stage Ta (B2) or in stage T1 (B3) and all colon tumours (C1). For each group of tumours, an average intensity value for the group was calculated for each SNP. From these, DNA copy numbers were estimated using a threshold of ±2 s.d. (above 2 s.d.: gain, below 2 s.d.: loss, and otherwise two copies, i.e. normal copy number). Subsequently, for each group of tumours, the genome was divided into regions according to the DNA copy numbers (gain, loss, normal) of consecutive SNPs. Genomic regions without any SNP data were not considered (see Supplementary Material for more information). Subsequently, it was investigated if the distribution of genomic copy number alterations correlated with the genomic distribution of 283 miRNAs (identified in the miRNA registry (release 7.1), Griffiths-Jones, 2004; Griffiths-Jones *et al*, 2006).

## RESULTS

Using the method and the predefined groups of tumours described in Materials and Methods, we identified commonly occurring copy number alterations in the groups. Genomic alterations are generally coherent with previous findings (Zieger *et al*, 2005; Andersen *et al*, 2006; Tørring *et al*, submitted).

### Location of miRNAs

**Figure 1:**
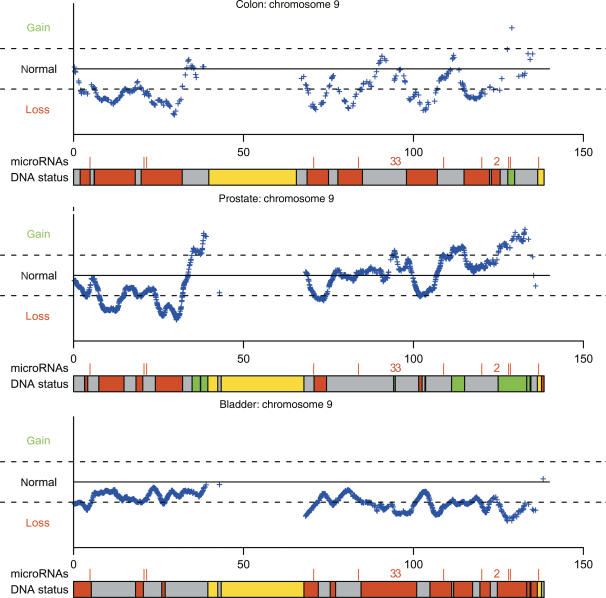
Definition of loss, normal and gain regions and location of the miRNAs, using chromosome 9 as an example. For each SNP, the average of the weighted signal intensities has been plotted. The dotted lines represent the cutoff of two standard deviations. The miRNA locations are denoted by a red line or a number. Each number (here 2 or 3) corresponds to one cluster and gives the number of miRNAs contained in that cluster. The DNA status bar summarises the information given by the plot. The red colour corresponds to the loss regions, the green to the gain regions, the grey to the normal regions and the yellow to the non-investigated regions (no SNPs available) (see Materials and Methods and the Supplementary Material).

The number of miRNAs affected by the alterations was counted and it was calculated if these numbers were unexpectedly high or low (Table 1 and Figure 1). For both the colon tumours (C1) and the prostate tumours (P1, P2 and P3), we observed an over-representation in gain regions and an under-representation of miRNAs in loss regions (*P*<0.00001) (Table 1 and Figure 1). As regards the bladder tumours (B1 and B3), we observed a slight under-representation of miRNAs in the gain regions (*P*=0.0089; Table 1).

Many miRNAs are located in close vicinity of each other (distance <0.1 Mb). After grouping miRNAs with distance <0.1 Mb into clusters, we ended up with 152 clusters and did the analysis on those. We found the same features as before (Table 1).

### Comparisons between the different tumours

We compared the different tumour groups in order to investigate whether the miRNAs were concentrated in loss or gain regions that were shared between the groups. Tables S1 and S2 in Supplementary Material clearly indicate that regions are generally not shared and that the findings above are not a consequence of shared alterations between tumour types.

### Genomic copy number alterations and miRNA expression

Volinia *et al* (2006) identified 39 overexpressed and six under-expressed miRNAs (out of 228) in a sample of prostate tumours and 21 overexpressed and one underexpressed miRNAs in a sample of colon tumours. The differentially expressed miRNAs were not statistically over-represented in the gain regions or loss regions identified in the corresponding tumours in our samples (*P*-values>0.18; see Table 2).

## DISCUSSION

In several papers, it has been shown that mRNA (or gene) expression and gene copy number correlate (e.g. Benetkiewicz *et al*, 2005; Tsafrir *et al*, 2006). It is natural to hypothesise that miRNA expression also correlates with copy number. In this study, we have reported on the location of miRNAs in relation to copy number changes in three different cancers, prostate, colon and bladder. In the prostate and colon tumours, we observed an over-representation in the gain regions and an under-representation of miRNA genes in the loss regions. Generally the gain and the loss regions were not shared between the two cancer types and miRNAs were generally not located in shared regions. This pattern is consistent with the hypothesis stated above and the findings in Volinia *et al* (2006) that many miRNA were overexpressed in prostate and colon tumours while few were underexpressed. However, when scrutinising the locations of the differentially expressed miRNAs it appears that these are not more (less) frequently located in gain (loss) regions; contrary to what we would expect if miRNA expression correlated with copy number. Importantly, we found a different pattern of copy number alterations in our set of bladder tumours, indicating that the relationship between cancer-related regions and miRNA locations is not straight-forward and probably cancer type specific.

Several explanations could accommodate for the discrepancies between expression and copy numbers. First of all, the copy numbers and the miRNA expressions values are obtained from different samples and thus could show different conflicting features. However, our samples as well as the samples in Volinia *et al* (2006) are not believed to represent special groups or subtypes of prostate and colon tumours, and general features should thus be preserved between the samples.

Secondly, one could raise doubt about the validity of the data. However, in a number of papers (Huang *et al*, 2004, 2006) it has been shown that copy numbers reliably can be derived using SNP arrays. The miRNA expression data set is one of a few public available genome-wide data sets; in the only other data set that is known to us (Lu *et al*, 2005) it is found that miRNA expression consistently is downregulated in prostate, while the pattern in colon is less clear.

Finally, miRNA expression could be regulated by mechanisms very different from the mechanisms that regulate mRNA expression, resulting in a less obvious pattern between expression and copy numbers. For example, miRNA expression could be correlated to the expression of their numerous mRNA targets (Griffiths-Jones, 2004), which might be located in different parts of the genome and thus potentially have different copy-numbers.

The data is available upon request.

## Figures and Tables

**Table 1:** Analysis of copy numbers in relation to miRNA location in seven different groups of tumours

	**Number of miRNAs**			**Number of miRNA clusters**		
	**Loss**	**Normal**	**Gain**	**Loss**	**Normal**	**Gain**
***C1 (colon)***						
**Obs.**	**66**	**172**	**38**	**47**	**72**	**28**
**Exp.**	**113.4**	**137.8**	**24.8**	**60.4**	**73.4**	**13.2**
**Size (in %)**	**41.1**	**49.9**	**9.0**	**41.1**	**49.9**	**9.0**
						
***P1 (prostate)***						
**Obs.**	**35**	**175**	**71**	**21**	**102**	**27**
**Exp.**	**89.2**	**156.9**	**34.9**	**47.6**	**83.8**	**18.6**
**Size (in %)**	**31.7**	**55.8**	**12.4**	**31.7**	**55.8**	**12.4**
						
*P2 (without metastasis)*						
Obs.	14	257	10	12	132	6
Exp.	37.9	240.4	2.7	20.2	128.4	1.4
Size (in %)	13.5	85.6	0.9	13.5	85.6	0.9
						
*P3 (with metastasis)*						
Obs.	28	115	138	16	92	42
Exp.	71.3	167.1	42.6	38.0	89.2	22.8
Size (in %)	25.4	59.5	15.2	25.4	59.5	15.2
						
***B1 (bladder)***						
**Obs.**	**42**	**228**	**11**	**33**	**111**	**6**
**Exp.**	**39.5**	**214.9**	**26.7**	**21.1**	**114.7**	**14.2**
**Size (in %)**	**14.1**	**76.5**	**9.5**	**14.1**	**76.5**	**9.5**
						
*B2 (stage Ta)*						
Obs.	7	274	0	5	145	0
Exp.	2.6	277.9	0.5	1.4	148.3	0.3
Size (in %)	0.9	98.9	0.2	0.9	98.9	0.2
						
*B3 (stage T1)*						
Obs.	33	235	13	28	114	8
Exp.	32.5	219.2	29.2	17.4	117.0	15.6
Size (in %)	11.6	78.0	10.4	11.6	78.0	10.4

Obs.: observed numbers of miRNAs in the three region types (loss, gain, normal); Exp.: expected numbers of miRNAs assuming the numbers are proportional to the size of the genome for a given region type; Size (in %): the size of the genome for a given region type in percentage.

7 miRNAs (five clusters) were excluded from the analysis of the colon tumours and 2 miRNAs (two clusters) were excluded for the prostate and bladder tumours because they were not located near any SNPs (see Supplementary Material). The three main groups are highlighted in bold.

*χ*^2^ test for proportionality to the size of the region: unclustered miRNAs. C1, P1-3: *P*<0.00001, B1: *P*=0.0089, B2: *P*=0.056, B3: *P*=0.0085. Clustered miRNAs. C1, P1-3: *P*<0.0006, B1: *P*=0.0054, B2: *P*=0.028, B3: *P*=0.010.

**Table 2:** Comparison between expression and location of miRNAs

	**Loss**	**Normal**	**Gain**
**Colon**			
*Overexpressed*			
Obs.	6	7	4
Exp.	4.1	10.6	2.3
			
*Underexpressed*			
Obs.	0	1	0
Exp.	0.2	0.6	0.1
			
**Prostate**			
*Overexpressed*			
Obs.	6	24	5
Exp.	4.4	21.8	8.8
			
*Underexpressed*			
Obs.	0	5	1
Exp.	0.7	3.7	1.5

Obs.: observed number of miRNAs; Exp.: expected number of miRNAs.

*P*-values above 18% for both cancer types.
