# Supplementary material for: Are microRNAs located in genomic regions associated with cancer?
Source: Br J Cancer. 2006 Sep 26;95(10):1415–8. doi: 10.1038/sj.bjc.6603381 (PMC2360608; doi:10.1038/sj.bjc.6603381)
Supplement: Supplementary Material 1 [file 95-6603381x1.doc]

**SUPPLEMENTARY MATERIAL**

**Lamy, Andersen, Dyrskjøt, Tørring, Ørntoft, Wiuf**

**Are miRNAs located in genomic regions associated with cancer?**

**CONTENT**

**Materials and Methods page 1**

**References page 4**

**Table S1 page 6**

**Table S2 page 7**

**Materials and Methods**

**Colon samples**

We used 15 samples from patients diagnosed with left sided colorectal adenocarcinomas and 113 normal samples collected at Aarhus University Hospital, Skejby. Of the 113 normal samples, 13 were blood samples from the same patients diagnosed with colorectal adenocarcinomas. The GeneChipMapping 10k Early Access Array was applied to all 128 samples. Experimental handling of the data is described elsewhere (Anderson *et al*, 2006). The array has 10,126 SNPs. Of these 8,452 (8,316 autosomal and 136 X chromosomal) mapped to a unique position in the genome (using the May 2004 genome assembly (hg17), [http://genome.ucsc.edu](http://genome.ucsc.edu/)). The remaining SNPs were excluded from the further analysis.

**Prostate and bladder samples**

For this second part of the study we used 41 samples from patients diagnosed with clinically verified prostate cancer, 30 samples from patients diagnosed with bladder cancer and 72 normal samples collected at Aarhus University Hospital, Skejby. Of the 41 samples from patients with prostate cancer, 15 were diagnosed without metastasis and 25 with metastasis. No information was available for the last one (Tørring *et al*, 2006). Of the 30 samples from patients with bladder cancer, 8 were in stage Ta and 22 were in stage T1. Of the 72 normal samples, 38 were blood samples from the same patients diagnosed with prostate cancer and 29 were blood samples from the same patients diagnosed with bladder cancer. The GeneChipMapping 100k array was applied to all 143 samples. Only the array probes for Xba I cleaved DNA was used. Experimental handling of the data is described elsewhere (Andersen *et al*, 2006; Tørring *et al*, 2006; Zieger *et al*, 2005). The array has 58,960 SNPs. Of these 58,494 (57,290 autosomal and 1,204 X chromosomal) mapped to a unique position (using the May 2004 genome assembly (hg17), http://genome.ucsc.edu). The remaining SNPs were excluded from the further analysis.

**Data analysis**

The Genechip DNA Analysis Software (GDAS) was used to extract probe intensities from the 10k arrays and GDAS 3.0.2 Patch Software was used for the 100k arrays. The probe intensities were normalized and a single signal value (the observed signal) for each SNP in each array was obtained using the software package dChip (http://www.dchip.org).

**Extraction of weighted signal intensities**

After normalization and extraction of signal values for the 128 10k-arrays and the 143 50k-arrays, the data was further normalized SNP-wise to allow comparison between different SNPs. Signal intensities cannot be compared directly because different SNPs have different physical properties. The normal samples were used as a reference, and the cancer samples were calibrated relatively to the normal samples. Specifically, the data was normalized SNP-wise using the mean and the standard deviation (sd) of the normal samples (113 for the 10k-arrays and 72 for the 50k-arrays): z_ij_ = (x_ij_ - mean_j_) / sd_j_ where x_ij_ is the observed signal of SNP j in sample i (normal or tumour), and mean_j_ and sd_j_ are mean and standard deviation of SNP j for all 113 normal samples for the 10K-arrays or all the 72 normal samples for the 50K-arrays. To further reduce the noise level in the signal values, we calculated the average of all SNPs within a 4 Mb region centred around SNP j weighted by genomic distance; ie a_ij_ = Σ (z_ik *_ exp(-d_jk_)) / ( Σ exp(-d_jk_)) where the sum (Σ) is over all the SNPs which are less than 2 Mb away from SNP j and d_jk_ is the genomic distance between SNP j and SNP k in Mb. The value a_ij_ is referred to as the weighted signal intensities. This procedure has been used elsewhere (Zieger *et al*, 2005; Anderson *et al*, 2006).

**Mapping of genomic regions commonly showing copy number alterations**

Genomic regions showing common copy number alterations were identified as regions where the average of the weighted signal intensities in a group of tumours (to be defined below) were above 2 standard deviations for regions of gain and under 2 standard deviations for regions of loss. The following groups were considered: the prostate (P1), bladder (B1) or colon tumours (C1), the prostate tumours without metastasis (P2) or with metastasis (P3) and the bladder tumours in stage Ta (B2) or in stage T1 (B3), as defined in the main text. Moreover, a position in the genome was not considered if there were no SNPs closer than 0.5 Mb for the 50k-array and 1.5 Mb for the 10k-array. As a consequence, the status of all (considered) positions in the autosomal chromosomes was either gain, normal or loss.

**The miRNA database**

A set of 283 miRNA genes were identified in the miR registry at <http://microrna.sanger.ac.uk/sequences/> (release 7.1). MiRNA genes located on the X chromosome were not included in this study. The miRNAs were mapped to a position in the genome using to the May 2004 genome assembly (hg17) at http://genome:ucsc.edu/.

**Clustering the miRNAs genes**

Many miRNAs genes are located in clusters. We defined two miRNAs to be in the same cluster if the distance between the first base of the first miRNA gene and the last base of the last miRNA gene of one cluster did not exceed 0.1 Mb. One cluster of miRNA was counted as one miRNA in the statistical analysis.

**Statistical analysis**

For each tumour type (bladder, prostate and colon) and for each subgroup (B2 and B3 for bladder and P2 and P3 for prostate), the total number of miRNAs and the total number of clusters were counted according to their position. They could be in a loss region, a normal (i.e. 2 copies) region, or a gain region. The regions were defined as explained previously. A chi-square test was carried out to test whether there is correlation between the locations of miRNAs and the tumour specific regions showing copy number alterations.

A comparison between the different tumour types and between the different sub-groups was performed in order to test whether the miRNAs were located mostly in regions where the tumour types/sub-groups showed similar copy number alterations. For each comparison, we defined the “loss” and the “gain” categories: the “loss” category corresponds to the genomic regions where one tumour is of loss and the other one is either of loss or normal while the “gain” category corresponds to the genomic regions where one tumour is of gain and the other one is either of gain or normal. We tested whether there was an over-representation of miRNAs in the common loss or gain regions within the “loss” or “gain” category.

**References**

Andersen CL, Wiuf C, Kruhøffer M, Korsgaard M, Laurberg S, Ørntoft TF. (2006) Genomic alterations of colorectal cancer identified and characterized by SNP- and expression array analysis. *Carcinogenisis*, Advanced access.

Tørring N, Borre M, Sørensen KD, Andersen CL, Wiuf C, Ørntoft TF. Genome-wide analysis of loss of heterozygosity (LOH) and copy number abnormalities in laser microdissected prostate cancer tissue using the Affymetrix 50K SNP mapping array identifies genotypes associated with metastasis and differentiation in prostate cancer. *Submitted*

Zieger, K., Dyrskjot, L., Wiuf, C., Jensen, J.L., Andersen, C.L., Jensen, K.M. & Orntoft, T.F. (2005) Role of Activating Fibroblast Growth Factor Receptor 3 Mutations in the Development of Bladder Tumors.*Clin Cancer Res,* **11**: 7709-7719, doi: 10.1158/1078-0432.CCR-05-1130.

**Table S1:** Comparison of the different tumour types in relation to miRNA location.

|  |  | **B1/P1** | **C1/P1** | **C1/B1** |  | **P2/P3** | **B2/B3** |
| --- | --- | --- | --- | --- | --- | --- | --- |
| **”Loss” category** | **Size (in %)** | **35.8** | **44.2** | **40.8** |  | **25.7** | **10.8** |
| *Normal/Loss* | Obs. | 26 | 12 | 21 |  | 3 | 26 |
|  | Exp. | 42.6 | 12.2 | 12.5 |  | 2.7 | 30.4 |
|  | Rel. size (in %) | 65.8 | 16.7 | 15.1 |  | 8.8 | 92.0 |
| *Loss/Loss* | Obs. | 9 | 21 | 15 |  | 11 | 7 |
|  | Exp. | 9.3 | 34.0 | 12.7 |  | 12.3 | 2.3 |
|  | Rel. size (in %) | 14.2 | 46.6 | 15.4 |  | 39.6 | 7.0 |
| *Loss/Normal* | Obs. | 30 | 40 | 47 |  | 17 | 0 |
|  | Exp. | 13.1 | 26.8 | 57.7 |  | 16.0 | 0.3 |
|  | Rel. size (in %) | 20.2 | 36.7 | 69.6 |  | 51.6 | 1.0 |
| **”Gain” category** | **Size (in %)** | **16.5** | **14.9** | **13.0** |  | **14.1** | **9.7** |
| *Normal/Gain* | Obs. | 64 | 56 | 6 |  | 0 | 13 |
|  | Exp. | 38.7 | 49.6 | 16.8 |  | 0.7 | 12.8 |
|  | Rel. size (in %) | 51.6 | 53.9 | 40.0 |  | 0.5 | 98.4 |
| *Gain/Gain* | Obs. | 4 | 9 | 1 |  | 10 | 0 |
|  | Exp. | 10.5 | 15.0 | 2.1 |  | 7.9 | 0.2 |
|  | Rel. size (in %) | 14.0 | 16.3 | 5.0 |  | 5.7 | 1.3 |
| *Gain/Normal* | Obs. | 7 | 27 | 35 |  | 128 | 0 |
|  | Exp. | 25.8 | 27.4 | 23.1 |  | 129.9 | 0.0 |
|  | Rel. size (in %) | 34.4 | 29.8 | 55.0 |  | 93.8 | 0.3 |
| **Other** | **Size (in %)** | **41.3** | **32.1** | **37.5** |  | **52.7** | **72.1** |
| *Normal/Normal* | Obs. | 138 | 104 | 145 |  | 112 | 235 |
|  | Rel. size (in %) | 96.3 | 94.1 | 91.5 |  | 100.0 | 100.0 |
| *Loss/Gain* | Obs. | 3 | 5 | 4 |  | 0 | 0 |
| *Gain/Loss* | Obs. | 0 | 2 | 2 |  | 0 | 0 |
|  | Rel. size (in %) | 3.7 | 5.9 | 8.5 |  | 0.0 | 0.0 |
| **Not considered** | **Size (in %)** | **7.4** | **8.8** | **8.8** |  | **7.4** | **7.4** |
| B1=bladder tumours; P1=prostate tumours; C1=colon tumours; P2=prostate tumours without metastasis; P3=prostate tumours with metastasis; B2=bladder tumours in stage Ta; B3=bladder tumours in stage T1.  Obs=observed number of miRNAs; Exp=expected number of miRNAs; Size (in %)=size of the genome for a given category type in percentage; Rel. size (in %)=size of the genome for a given region type in percentage (100% is the size of the genome for the category). E.g., the “*Normal/Loss*” subgroup contains the regions of the genome which are normal in the first tumour type and loss in the second.  When comparing observed and expected numbers, it appears that the majority of miRNAs are not located in shared loss or gain regions (loss/loss or gain/gain). | | | | | | | |

**Table S2:** Comparison of the different tumour types in relation to miRNA clusters location.

|  |  | **B1/P1** | **C1/P1** | **C1/B1** |  | **P2/P3** | **B2/B3** |
| --- | --- | --- | --- | --- | --- | --- | --- |
| **”Loss” category** | **Size (in %)** | **35.8** | **44.2** | **40.8** |  | **25.7** | **10.8** |
| *Normal/Loss* | Obs. | 13 | 5 | 16 |  | 2 | 23 |
|  | Exp. | 28.2 | 8.2 | 9.2 |  | 1.6 | 25.8 |
|  | Rel. size (in %) | 65.8 | 16.7 | 15.1 |  | 8.8 | 92.0 |
| *Loss/Loss* | Obs. | 8 | 15 | 13 |  | 10 | 5 |
|  | Exp. | 6.1 | 22.8 | 9.4 |  | 7.1 | 2.0 |
|  | Rel. size (in %) | 14.2 | 46.6 | 15.4 |  | 39.6 | 7.0 |
| *Loss/Normal* | Obs. | 22 | 29 | 32 |  | 6 | 0 |
|  | Exp. | 8.7 | 18.0 | 42.4 |  | 9.3 | 0.3 |
|  | Rel. size (in %) | 20.2 | 36.7 | 69.6 |  | 51.6 | 1.0 |
| **”Gain” category** | **Size (in %)** | **16.5** | **14.9** | **13.0** |  | **14.1** | **9.7** |
| *Normal/Gain* | Obs. | 22 | 16 | 3 |  | 0 | 8 |
|  | Exp. | 14.4 | 23.2 | 11.6 |  | 0.2 | 7.9 |
|  | Rel. size (in %) | 51.6 | 53.9 | 40.0 |  | 0.5 | 98.4 |
| *Gain/Gain* | Obs. | 2 | 7 | 1 |  | 6 | 0 |
|  | Exp. | 3.9 | 7.0 | 1.4 |  | 2.4 | 0.1 |
|  | Rel. size (in %) | 14.0 | 16.3 | 5.0 |  | 5.7 | 1.3 |
| *Gain/Normal* | Obs. | 4 | 20 | 25 |  | 36 | 0 |
|  | Exp. | 9.6 | 12.8 | 15.9 |  | 39.4 | 0.0 |
|  | Rel. size (in %) | 34.4 | 29.8 | 55.0 |  | 93.8 | 0.3 |
| **Other** | **Size (in %)** | **41.3** | **32.1** | **37.5** |  | **52.7** | **72.1** |
| *Normal/Normal* | Obs. | 76 | 51 | 53 |  | 90 | 114 |
|  | Rel. size (in %) | 96.3 | 94.1 | 91.5 |  | 100.0 | 100.0 |
| *Loss/Gain* | Obs. | 3 | 2 | 2 |  | 0 | 0 |
| *Gain/Loss* | Obs. | 0 | 2 | 2 |  | 0 | 0 |
|  | Rel. size (in %) | 3.7 | 5.9 | 8.5 |  | 0.0 | 0.0 |
| **Not considered** | **Size (in %)** | **7.4** | **8.8** | **8.8** |  | **7.4** | **7.4** |
| B1=bladder tumours; P1=prostate tumours; C1=colon tumours; P2=prostate tumours without metastasis; P3=prostate tumours with metastasis; B2=bladder tumours in stage Ta; B3=bladder tumours in stage T1.  Obs=observed number of miRNA clusters; Exp=expected number of miRNA clusters; Size (in %)=size of the genome for a given category type in percentage; Rel. size (in %)=size of the genome for a given region type in percentage (100% is the size of the genome for the category). E.g., the “*Normal/Loss*” subgroup contains the regions of the genome which are normal in the first tumour type and loss in the second.  When comparing observed and expected numbers, it appears that the majority of miRNA clusters are not located in shared loss or gain regions (loss/loss or gain/gain). | | | | | | | |
